# Molnupiravir Outpatient Treatment for Adults with COVID-19 in a Real-World Setting—A Single Center Experience

**DOI:** 10.3390/jcm11216464

**Published:** 2022-10-31

**Authors:** Kinga Czarnecka, Paulina Czarnecka, Olga Tronina, Magdalena Durlik

**Affiliations:** Department of Transplant Medicine, Nephrology and Internal Diseases, Medical University of Warsaw, 59 Nowogrodzka Street, 02-006 Warsaw, Poland

**Keywords:** molnupiravir, COVID-19, chronic kidney disease, renal transplant

## Abstract

Background: Molnupiravir is approved for the treatment of adult patients with mild to moderate COVID-19. The main goal of the treatment is to reduce hospitalization and mortality rate. This study aimed at the all-cause hospitalization and all-cause death assessment in patients at high risk of severe COVID-19 treated with molnupiravir. Methods: This was a prospective, observational single center study. Non-hospitalized patients with SARS-CoV-2 infection, COVID-19 symptoms with the onset of up to 5 days, and at high risk of severe COVID-19 illness received molnupiravir based on attending physician decisions. Results: In total, 107 patients were enrolled. Adverse events were reported in 28.0% of patients, with nausea and abdominal pain being the most commonly observed. No treatment-emergent AEs resulted in therapy discontinuation. Overall, 15 patients required hospitalization. During the observation, 2.8% (*n* = 3) of patients subsequently died. All deaths were considered to be related to COVID-19 complications. Age over 65 years, heart failure, and ischemic heart disease showed a significant correlation with the severe course of COVID-19. Conclusion: Molnupiravir may be perceived as an alternative treatment for patients with immunosuppression and advanced chronic kidney disease. Nevertheless, further studies are required to conclusively establish a role for molnupiravir in future COVID-19 treatment recommendations.

## 1. Introduction

### Background

The coronavirus disease 2019 (COVID-19) pandemic, caused by the severe acute respiratory syndrome coronavirus 2 (SARS-CoV-2), has affected almost 515 million people and resulted in over 6.2 million deaths worldwide [1]. Several vaccines against SARS-CoV-2, which proved to be highly effective in preventing hospitalization and death risk reduction, were expected to be game changers during the pandemic. However, this preventive measure turned out to be insufficient. On the one hand, there is the hesitancy exhibited by many individuals against the vaccination, and on the other hand, is the reduced efficacy of vaccines in preventing severe COVID-19 in certain subpopulations (e.g., organ transplant recipients, patients on dialysis) [2,3,4]. Moreover, SARS-CoV-2, an RNA virus, exerts mutagenic potential in the evolutionary adaptation process, which results in the emergence of new genetic SARS-CoV-2 variants with altered antigenicity of the spike protein, some of which carry immune escape mutations [5]. Therefore, reduced effectiveness of antibody-mediated immunity triggered by vaccination and the uncertain effectiveness of currently available anti-SARS-CoV-2 pharmacological agents may be expected [6]. Therefore, there is a need to develop safe and effective treatments to prevent severe COVID-19.

Molnupiravir (EIDD-2801/MK-4482) is a newly developed prodrug that exhibits broad-spectrum antiviral activity against many RNA viruses, including the new SARS-CoV-2 virus. The drug is approved for the treatment of adult patients with mild-to-moderate COVID-19 in the pre-hospital phase of infection (up to 5 days after symptom onset) with at least one risk factor for severe illness. 

Molnupiravir was the first orally administered drug against SARS-CoV-2 available on the market. The initial results of molnupiravir administration were promising, and the drug was found to reduce the hospitalization rate and the risk of death by 50% [7]. However, subsequent studies have not confirmed these results, documenting approximately a 30% success rate in reducing hospitalization and death rates [8].

At the beginning of this study, molnupiravir remained the only drug approved to prevent the severe course of COVID-19 infection in the outpatient population. Despite greater availability of current COVID-19 agents on the market, given their extensive drug–drug interactions, mostly intravenous routes of administration and restrictions for use, molnupiravir may still constitute a useful alternative for a subset of patients.

## 2. Materials and Methods

### 2.1. Study Population and Design

This single-center observational study was conducted in the transplant medicine and nephrology outpatient clinic and hemodialysis center of the Department of Transplant Medicine, Nephrology, and Internal Diseases, Medical University of Warsaw, between 18 January 2022 and 31 March 2022. Adult subjects, who provided written informed consent, were enrolled in this study. Administration of the treatment regimen was at the discretion of the attending physician based on the current knowledge and local recommendations of the Polish Association of Epidemiologists and Infectiologists (PTEiLChZ) [9]. The diagnosis of COVID-19 was determined by reverse-transcriptase polymerase chain reaction (RT-PCR) or antigen testing using a nasopharyngeal swab. Non-hospitalized patients with laboratory-confirmed mild-to-moderate SARS-CoV-2 infection, COVID-19 symptoms with the onset of up to 5 days before potential molnupiravir administration, and at high risk of severe COVID-19 illness (defined as the presence of at least one of the risk factors presented in Table 1 [10]), were eligible for the study. To avoid the duplication of a number of risk factors, subjects who underwent kidney transplants were not considered for a chronic kidney disease risk factor. This rule did not apply to transplant recipients of organs other than kidneys. SARS-CoV-2 infection severity was determined based on the definition provided by the Food and Drug Administration [11]. Vaccinated and unvaccinated individuals were included in this study. Patients with medical indications for hospital admission at the baseline were excluded. Molnupiravir was initiated within 5 days (all inclusive) of symptom onset and continued for 5 consecutive days at a dose of 800 mg (4 tablets) at 12 h intervals. For solid organ transplant recipients (SOTRs), at COVID-19 diagnosis and initiation of molnupiravir, the immunosuppressive regimen was modified. Patients on the triple immunosuppressive regimen were instructed to withhold mycophenolate mofetil (MMF) for five consecutive days, after which MMF was reintroduced. The doses of the remaining immunosuppressants were maintained. In patients who required hospitalization, further immunosuppressive therapy was modified depending on their current clinical condition, risk of organ rejection, and concurrent complications of the underlying disease. Molnupiravir was the only COVID-19 dedicated treatment administered to the patients in the study. Individuals who received any additional COVID-19 dedicated treatment were excluded, as were patients receiving antibiotic therapy, in order to reduce the number of confounding factors during the analysis. Symptomatic management of COVID-19 (including, but not limited to, antipyretic drugs) was permitted. Information was obtained regarding the patients’ vaccination status and medical history of previously documented SARS-CoV-2 infection. Information regarding adverse events (AEs) was collected during the treatment period and for 30 consecutive days following treatment cessation. Additionally, data on COVID-19 symptoms and baseline oxygen saturation levels measured by pulse oximetry were obtained during medical consultation. Compliance with the ordered treatment and dosage regimen was monitored based on the information provided by the patients via telephone. Hospitalized patients were followed up for the final outcome, the requirement of oxygen supply, or mechanical ventilation. 

The study was conducted in accordance with the provisions of the Declaration of Helsinki and received a favorable local ethics committee opinion.

### 2.2. Statistical Analysis

The normality of continuous variables was tested using the Shapiro–Wilk test. The relationship between two nominal factors was investigated using Fisher’s exact test. When examining the dependence of two dichotomous variables (*df* = 1), the phi Yule coefficient *φ_c_* was reported as a measure of association. For variables with a greater degree of freedom (*df* ≥ 2), the Cramer *V* measure of association was used.

When examining the differences in a non-normally distributed continuous variable between two groups, the Mann–Whitney *U* test was used with a related measure in the form of ranked biserial correlation, ${\hat{r}}_{biserial}^{rank}$. This measure was also used to study the correlation between ordinal and nominal values. 

Welch’s *t*-test was used to test the differences in a normally distributed continuous scale between the two variable groups, and the size of the effect was estimated using Hedges’ *g*.

Analyses were conducted using the R statistical language (version 4.1.1) [12] on Windows 10 × 64 (build 19044) using the packages rstatix (version 0.7.0) [13], effect size (version 0.6.0.1) [14]), sjPlot (version 2.8.9) [15], report (version 0.5.1) [16], ggstatsplot (version 0.9.0) [17] psych (version 2.1.6) [18], and rcompanion (version 2.4.13) [19].

### 2.3. Aim and Study Endpoints

The aim of this study was to assess the risk of all-cause hospitalization and all-cause death in patients at high risk of severe COVID-19 treated with molnupiravir. The primary endpoints were all-cause hospitalization and all-cause death. Hospitalization was defined as at least 24 h hospital stay. The secondary endpoint was determined based on the safety assessment of molnupiravir treatment and defined as observed AEs, adverse event-related therapy interruption, or withdrawal.

## 3. Results

The study population comprised 107 patients, 65 of whom were men. Characteristics of the study population are presented in Table 1. The study population had a mean age of 53.7 years (standard deviation (SD) = 18.1). Of 107 patients, 86.0% (*n* = 92) and 14% (*n* = 15) tested positive based on the RT-PCR and antigen tests, respectively. Previous history of COVID-19 was reported in 11.2% (*n* = 12) of participants. Of 83 patients (77.6%) who completed the full anti-SARS-CoV-2 vaccination course, 78 (72.9%) received an mRNA vaccine, and 56 (67.5%) received a booster dose. 

Approximately half (43.9% (*n* = 47)) of participants had two risk factors for severe COVID-19 illness, followed by three and four risk factors identified in 26.1% (*n* = 28) and 18.7% (*n* = 20), respectively. The distribution of risk factors are shown in Table 1.

The predominant risk factor was immunosuppressive treatment, identified in 67.3% (*n* = 72) of participants, followed by chronic kidney disease (42.8% (*n* = 49)) (Table 1). Among the 72 immunocompromised patients, 71 were SOTRs. Most transplant recipients (*n* = 58) received a triple immunosuppressive regimen (steroids, MMF, and tacrolimus). After transplantation, 60 of 71 patients were fully vaccinated, and 43 received a booster dose; similarly, 35 patients with chronic kidney disease were fully vaccinated, and 25 received a booster. 

The most commonly reported clinical symptoms among the participants were cough and fever reported in over 50% of patients, followed by a sore throat and muscle pain in 33.7% and 17.8% of patients, respectively. Dyspnea was reported in only 4.6%. COVID-19 symptoms and the frequency of their occurrence are presented in Table 2.

The mean time from COVID-19 symptom onset to the first molnupiravir dose was 3.15 days (SD = 1.29; range 0–5 days). Overall, 15 patients required hospitalization. Oxygen supply by nasal prongs, simple mask, or mask with reservoir was required in nine cases, two of which required high-flow oxygen supplementation. None of the patients admitted to the hospital were intubated or placed on mechanical ventilation. During the observation, 2.8% (*n* = 3) of patients who subsequently died were over 60 years of age, were unvaccinated, had at least four risk factors for severe COVID-19, and had no documented history of previous COVID-19 infection. All deaths occurred during the hospital stay and were considered to be related to COVID-19 complications as assessed by the treating physicians. The first patient died of myocardial infarction with ST-segment elevation 23 days after the cessation of molnupiravir treatment. The subject was initially at high risk of cardiovascular events due to his comorbidities (diabetes mellitus, chronic kidney disease on dialysis, and cardiovascular conditions). Despite the significant predisposition to cardiovascular events presented by the subject, treating physicians considered fatal myocardial infarction as COVID-19 complication-related, as well-documented and adequately controlled cardiovascular risk factors were highly unlikely to cause the event by themselves without COVID-19 co-occurrence. Respiratory insufficiency was reported as the cause of death in the second patient, who was in poor general condition at baseline and had underlying metastatic hepatocellular carcinoma that qualified for palliative care and was not eligible for further therapy escalation. The death of the third patient was due to cardiac arrest, unresponsive to resuscitation maneuvers. She was at an advanced age and had multiple comorbidities, including cardiovascular conditions, stage 4 of chronic kidney disease, and diabetes mellitus. Again, in the opinion of the treating physician, these conditions themselves were highly unlikely to trigger cardiac arrest at this point in time in the absence of symptomatic SARS-CoV-2 infection. Fatal outcomes were reported in the second and third cases 11 and 6 days after therapy completion, respectively. 

Among the hospitalized patients, 53.3% (*n* = 8) who were SOTRs were all vaccinated, and seven received a booster. The median time after organ transplant was 6.6 (SD = 7.0; range 0–29.8) years. Only four patients required oxygen therapy, and none of the SOTRs died. 

Equally, eight patients with chronic kidney disease required hospital admission, three of whom additionally had active cancer; five were vaccinated, four received a booster, six required an oxygen supply, and three died. 

All the study participants completed a full course of molnupiravir treatment.

### 3.1. Relationship between the Pre-Defined Risk Factors and Severe COVID-19 Course

Age over 65 years, heart failure, and ischemic heart disease showed a significant correlation with increased risk of hospitalization and death (see Table 3).

There was a significant relationship between the number of risk factors and hospitalization risk (*p_Fisher_* < 0.001, *df* = 5, *V* = 0.53). Hospitalization risk in patients with two risk factors accounted for 3.7% and increased to 5.6% with five risk factors (*p_Fisher adj_* = 0.001, *V* =0.61). A corresponding correlation was observed between patients with three and five risk factors (*p_Fisher adj_* = 0.001, *V* = 0.75).

A similar relationship was observed between the number of risk factors and death (*p_Fisher_* = 0.015, *df* = 5, *V* = 0.40). Thus, significant differences were found between the patients’ probability of death for risk factors two and five (*p_Fisher_ =* 0.019, *V* = 0.47), and between three and five (*p_Fisher_ =* 0.044, *V* = 0.45).

Individuals saturating < 96% at baseline were more often hospitalized (*M* = 95.53%, *SD* = 2.13%, n =15) compared to patients not requiring hospitalization (*M* = 97.00%, *SD* = 1.42%, n = 92), *t_Welch_* (16.09) = 2.57, *p* = 0.020, ${\hat{g}}_{Hedges}$ = 0.77).

A similar correlation was found for patients who died (*M* = 94.33%, *SD* =0.58, *n* = 3) versus patients who survived (*M* = 96.87%, *SD* = 1.58%, *n* = 104), *t_Welch_* (2.95) = 6.89, *p* = 0.007, ${\hat{g}}_{Hedges}$ = 1.53).

Neither a completed full course of vaccination against SARS-CoV-2, including receiving a booster dose, nor a previously documented history of COVID-19 infection showed to be preventative in hospitalization or death risk reduction (see Table 4).

There was a weak relationship between disease symptom onset time, molnupiravir treatment initiation time, and the risk of hospitalization (*V* = 0.27). When the molnupiravir was administered within 2 days of the symptom onset, the likelihood of hospitalization was 9.4%, whereas the value increased to 20.0% when molnupiravir was administered thereafter. However, these differences were not statistically significant (*p_Fisher_* = 0.138, *df* = 5). No significant correlation was found between the time of molnupiravir administration and the risk of death.

### 3.2. Analysis of Subpopulations

The type of transplanted organ and type of immunosuppressive regimen was insignificant in terms of hospitalization and death risk reduction (*p_Fisher_* = 1.000_,_ *df* = 5; *p_Fisher_* = 0.429, *df* = 8). Similarly, time after transplant demonstrated no correlation with the risk of hospital admission and death (*W*_Mann–Whitney_ = 235.50, *p* = 0.771, ${\hat{r}}_{biserial}^{rank}$ = −0.07, *n* = 71).

The stage of chronic kidney disease did not affect the course of infection (*df* = 3, *p_Fisher_* = 0.948, *V* = 0.13). 

### 3.3. Safety Profile

Adverse events were reported in 28.0% (*n* = 30) of patients, and the most commonly reported AEs, nausea and abdominal pain, were observed in 7.5% (*n* = 8) and 4.7% (*n* = 5) of patients, respectively. The majority of AEs were mild and did not require medical intervention. No treatment-emergent AEs resulted in therapy discontinuation. SOTRs were more likely to experience weakness and nausea. Nausea was a common side effect in patients with chronic kidney disease. Dizziness was more prominent in patients aged over 65 years, similar to individuals with diabetes mellitus. No allograft biopsies were clinically indicated during the study, as none of the SOTRs were suspected of having allograft rejection. Detailed information on all AEs according to risk group for severe COVID is presented in Table 5.

## 4. Discussion

To date, data on the administration of molnupiravir are limited. There is a need for larger and more inclusive studies that would enable thorough efficacy assessment, especially in populations that might be particularly vulnerable to severe COVID-19.

Scientific information or published medical literature regarding treatment with molnupiravir is scarce. Additionally, special subpopulations with an increased risk of developing severe COVID-19 are often excluded from studies. Unlike other currently published research, our study population included immunocompromised subjects, often excluded from COVID-19-dedicated trials despite their greater risk of severe course of infection compared to the general population. Additionally, our study included patients with chronic kidney disease, regardless of the stage of the disease, and vaccinated individuals. According to local sources, at the time of conducting this study, Omicron (B. 1. 1. 529), which is known to be less virulent compared to previously identified variants, was the dominant variant of SARS-CoV-2, and it accounted for over 70% of infection in all country regions [20]. These factors may have significantly impacted the study results.

Reported hospitalization and death rates were low among the study population accounting for 14% and 2.8%, respectively. Even though the effectiveness of the molnupiravir treatment is infeasible to assess due to the lack of a formal control group enrolled, our results appear to be in keeping with the previously published outcomes of clinical trials which documented molnupiravir effectiveness in preventing severe COVID-19 defined as a need for hospital admission or death [7,8]. Additionally, the most recent analysis of molnupiravir effectiveness in the real-world setting during SARS-CoV-2 Omicron variant dominance both in the general population and in STORs supports our findings [21,22]. Despite this, the study by Flisiak et al. did not take into account subjects’ vaccination status and previous SARS-CoV-2 infection, and Radcliff’s paper did not offer data regarding adherence to molnupiravir treatment.

Risk factors that were found to be associated with increased risk of hospitalization and death were age over 65 years, heart failure, and ischemic heart disease. This is in line with the results obtained from other studies that identified older age and cardiovascular comorbidities as risk factors for severe disease [23].

Not only the risk factor type but also a number of risk factors for severe COVID-19 significantly impacted both the risk of hospitalization and death. Fatal outcomes were reported only in subjects with four or more risk factors for severe COVID-19. In addition, patients who showed a lower baseline oxygen saturation status were more likely to be hospitalized or die. These findings are consistent with those of previous studies. Pereira et al., in their COVID-19 SOTRs study, observed a relationship between initially lower oxygen saturation and the risk of hospital admission [24]. Corresponding results were noted in the general population [25,26]. Thus, baseline health condition and oxygenation status have a crucial impact on therapy outcomes and appear to be of paramount importance in predicting the risk of death or hospital admission. This underpins the need for early SARS-CoV-2 infection detection, optimization of coexisting comorbidities, and strict clinical monitoring from the date of diagnosis, with regular oxygen saturation measurements. Furthermore, our study indicates that participants with four or more risk factors for severe COVID-19 illness need to be more closely monitored for any potential deterioration in their health status if not hospitalized, considering the much greater risk of COVID-19-related complications and death. This study showed that baseline oxygen saturation might have a significant impact on the risk of hospitalization and death. Therefore, apart from oxygen saturation levels <94%, which is a universally acknowledged indication for hospital admission, patients with oxygen saturation <96% should also remain under close surveillance.

Neither the full course of vaccination nor the booster dose prevented the risk of hospital admission and death. This finding is not in agreement with previously published studies that have documented that available anti-SARS-CoV-2 vaccines are highly effective in preventing severe COVID-19 [27]. However, the specific study population and the emergence of new genetic SARS-CoV-2 variants may explain the discrepant outcomes of our research. Importantly, almost 70% of our study population included immunocompromised patients who are known to have impaired immunological response to vaccination. Based on the most recent meta-analysis, immunocompromised patients, especially after organ transplantation, are significantly less likely to show seroconversion rates after COVID-19 vaccination, with only one-third achieving this goal [4,28]. Although our study showed no association between vaccination status and COVID-19 severity, all fatal outcomes occurred in unvaccinated individuals. Taking into account that information pertaining to COVID-19 antibody levels was not gathered for the purpose of this study, as well as the low sample size of the study, caution should be exercised while utilizing the results of our analysis in terms of assessment of vaccination efficacy.

A correlation, though not statistically significant, was found between COVID-19 symptom onset, molnupiravir administration, and risk of hospital admission. The outcome was not statistically significant and could have been affected by the great number of vaccinated patients in the study population combined with the dominant SARS-CoV-2 variant at the time of the study. This may suggest that subjects at high risk of a severe course of the disease may benefit from the early initiation of molnupiravir treatment, and delay in treatment implementation should be avoided. Considering the mechanism of action of molnupiravir, which interferes with SARS-CoV-2 replication, medication is expected to be the most effective at the early stage of infection. However, the results obtained from our study dominated by immunocompromised individuals may not be easily translated to the general population. Further studies on large populations are needed to confirm this initial suggestion and to better define the optimal time for treatment initiation.

Organ transplant recipients are commonly recognized as a high-risk group for severe COVID-19, as worse outcomes have been reported for them compared to the general population [29]. Our study found no correlation between the type of transplanted organ or immunosuppressive regimen, time after organ transplant, and severe COVID-19 course. The reason for this may be that kidney transplant recipients and triple immunosuppressive regimens consisting of steroids, MMF, and tacrolimus were overrepresented in the study population in comparison to other organ recipients or other immunosuppressive regimens. Among the immunocompromised participants, only a small portion required hospitalization (11.3% (*n* = 8)), and none required mechanical ventilation or died. Our findings correspond to the results of Pereira and Favà, who documented no correlation between COVID-19 severity and baseline immunosuppression, type of transplanted organ, or time from transplantation. However, Pereira’s study population was also dominated by kidney transplant recipients, constituting over 50% of the whole, and Favà et al. included only inpatient kidney recipients [24,30]. Moreover, our findings cannot be directly compared to Pereira et al. and Favà et al. because dyspnea is one of the most common symptoms reported among their study populations, which is also a significant indicator of a severe COVID-19 course. In our study population, dyspnea was manifested in only 4.6% of cases. Favà et al. also excluded non-hospitalized patients. Further studies with more diverse organ transplant populations are needed to conclusively establish the relationship between transplant factors and the risk of severe COVID-19.

Chronic kidney disease is a risk factor for severe COVID-19. The European Renal Association − European Dialysis and Transplant Association (ERA-EDTA) Registry indicated that patients receiving renal replacement therapy are at an increased risk of COVID-19-related death [29]. However, no confirmed link was found between the initial stage of chronic kidney disease (G1/G2) and a greater risk of developing severe COVID-19 [27]. Conversely, we found no significant correlation between the chronic kidney disease stage and the severe course of COVID-19. These discrepant results may stem from the relatively small number of participants with chronic kidney disease and stage 2 of the disease present in over 20% of the whole chronic kidney disease group.

Patients with advanced chronic kidney disease or on dialysis are often excluded from clinical trials due to safety reasons, considering that the stage of chronic kidney disease may potentially influence COVID-19 outcomes. Given that according to the OpenSAFELY study, organ transplant recipients, patients with chronic kidney disease, and those receiving renal replacement therapy constitute three out of four subpopulations associated with the highest COVID-19-related mortality, ERA-EDTA calls for more inclusive studies [29,31,32]. To the best of our knowledge, this is the first published COVID-19 study that included both organ transplant recipients and patients with chronic kidney disease regardless of its stage. In our study, molnupiravir presented a favorable safety and tolerability profile in a population with at least one risk factor for a severe COVID-19 course, with a predominance of immunocompromised subjects. No safety concerns were identified during the study. AEs reported for the drug are also frequently observed in COVID-19 patients never exposed to molnupiravir; hence, it is challenging to distinguish whether the reported events are molnupiravir-related or are signs/symptoms of the condition under treatment. Arribas et al. faced the same challenge [33]. Fatal outcomes were noted only in subjects with four or more risk factors of severe illness and concomitant comorbidities and age > 60 years. All deaths were considered to be associated with COVID-19 complications. Other molnupiravir-related studies have confirmed these results, adding a beneficial pharmacokinetic profile to the list of molnupiravir assets [7,34,35]. Molnupiravir is not expected to require dose adjustment in patients with chronic kidney disease or in those receiving renal replacement therapy. Furthermore, it is not expected to disrupt metabolic pathways for medications metabolized by CYP450, nor does it interfere with drug transporter levels. This is of particular importance in patients on immunosuppressive medications, many of which are strong inhibitors of CYP3A4 and impact drug transporters levels, thus, increasing the risk of drug–drug interactions and related AEs.

According to the most recently issued guidelines on the therapeutic management of non-hospitalized adults with COVID-19, nirmatrelvir/ritonavir and remdesivir are recommended as the preferred therapies. Molnupiravir is listed as an alternative treatment option only when any of the preferred therapies are not available, feasible for use, or appropriate from a clinical perspective [36]. Corresponding recommendations have been issued for the transplant population, listing monoclonal antibodies and intravenous remdesivir as superior to molnupiravir in COVID-19 management [37]. However, significant limitations of use were identified for all first-line therapy options. Ritonavir is a potent inhibitor of CYP3A4, which is the main cytochrome responsible for drug metabolism, and it increases the risk of drug–drug interactions and related AEs. As such, combination products of nirmatrelvir/ritonavir may be of limited use in any individual receiving calcineurin inhibitors or mTOR inhibitors hence in most of the transplant population. Accordingly, nirmatrelvir/ritonavir administration in organ recipients is only suggested after appropriate dose adjustment of immunosuppressive medications and the feasibility of their serum levels monitoring during treatment. Furthermore, its use is not advised in patients with advanced stages of chronic kidney disease with a GFR < 30 mL/min as well as in patients with severe impairment of hepatic function. Keeping in mind that this therapeutic approach is intended for patients at high risk of severe COVID-19 illness, who are likely to have multiple comorbidities and be on many concomitant medications, weighing against therapeutic goals and potential risks of nirmatrelvir/ritonavir use may be challenging in clinical practice. The question about the potential SARS-CoV-2 resistance following nirmatrelvir/ritonavir exposure is yet to be answered, as cases of viral resistance have been documented in the past for other 3C protease inhibitors [38].

Remdesivir, on the other hand, must be administered for 3 consecutive days via intravenous infusion at the medical facility, enabling patients to be observed and managed for potentially severe hypersensitivity reactions for at least an hour after the infusion or longer if clinically justified. Similar precautions apply for monoclonal antibody administration.

The limitation of this study included its small and heterogeneous sample size, which yielded a significantly disproportionate representation of the study participants in each risk group. Considering that most of the participants were SOTRs, the lack of information on the participant’s immunological status, namely the level of anti-SARS-CoV-2 antibodies, may be a limiting factor in this study. The association of the prevalent SARS-CoV-2 variant in Poland during the study period, the increased number of vaccinated individuals in the general population, and the increasing proportion of individuals with a past medical history of COVID-19 with molnupiravir efficacy remains unknown. Additionally, Omicron is known to be less virulent than previously identified variants, and it manifests itself predominantly with mild respiratory and gastrointestinal symptoms, with rare, documented cases of severe respiratory insufficiency requiring hospitalization, ventilatory support, or fatal outcomes. The inclusion of vaccinated patients may also be perceived as another limitation. However, their inclusion in the study population better reflects the real-world population.

## 5. Conclusions

In conclusion, our study showed that molnupiravir is safe and may constitute an alternative for certain subpopulations not eligible for the recommended first-line therapies, including immunocompromised patients and those with advanced-stage chronic kidney disease or hepatic injury. Nevertheless, further studies are required to conclusively establish a role for molnupiravir in future COVID-19 treatment recommendations.

## Figures and Tables

**Table 1:** Baseline characteristics of the study population.

Characteristics	Value [*N* = 107]
Age *	
mean	53.7 (18.1) years
range	24–95 years
Male sex	65 (60.75%)
Risk factors for severe COVID-19	
Immunosuppression	72 (67.3%)
Transplantation	71 (66.4%)
Type of transplanted organ	
kidney	51 (71.8%)
liver	13 (18.3%)
Other **	7 (9.6%)
Time after transplant *	6.6 (7.0) years
Age > 65 years	28 (26.2%)
Diabetes mellitus	34 (31.8%)
Obesity	1 (0.9%)
Chronic obstructive pulmonary disease	3 (2.8%)
Chronic kidney disease	49 (42.8%)
stage 2	11 (22.5%)
stage3	19 (38.8%)
stage 4	5 (10.2%)
stage 5	14 (28.6%)
Heart failure	16 (14.9%)
Ischemic heart disease	24 (22.5%)
Cardiomyopathy	-
Nursing home resident	-
Active cancer	9 (8.4%)
Subjects with two risk factors for severe COVID-19	47 (43.9%)
Subjects with three risk factors for severe COVID-19	28 (26.1%)
Subjects with four risk factors for severe COVID-19	20 (18.7%)
Subject with more than four risk factors for severe COVID-19	9 (8.4%)
SARS-CoV-2 infection confirmation	
RT-PCR test	92 (86.0%)
Antigen test	15 (14.0%)
Confirmed previous history of COVID-19	12 (11.2%)
Vaccination status	
Full course of vaccination completed	83 (77.6%)
Received booster dose	56 (67.5%)

* Mean ± SD. All other values are n (%). ** “Other” indications included lung transplant (2 cases), kidney and pancreas transplant (2 cases), pancreas transplant. (1 case), kidney and liver transplant (1 case), heart transplant (1 case).

**Table 2:** Clinical symptoms of COVID-19 and the frequency of occurrence.

Clinical Symptoms	Frequency (%)	
	Yes	No
Cough	58 (54.2%)	49 (45.8%)
Fever	56 (52.4%)	51 (47.6%)
Sore throat	36 (33.7%)	71 (66.3%)
Myalgia	19 (17.8%)	88 (82.2%)
Headache	15 (14%)	92 (86%)
Subfebrile state	15 (14%)	92 (86%)
Weakness	15 (14%)	92 (86%)
Diarrhea	10 (9.3%)	97 (90.6%)
Nausea	8 (7.5%)	99 (92.5%)
Lack of appetite	8 (7.5%)	99 (92.5%)
Fatigue	7 (6.5%)	100 (93.5%)
Vomiting	6 (5.6%)	101 (94.4%)
Dyspnea	5 (4.6%)	102 (95.3%)
Runny nose	5 (4.7%)	102 (95.3%)
Sinusitis	5 (5.6%)	101 (94.4%)
Nasal congestion	4 (3.7%)	103 (96.2%)
Other	14 (13.1%)	93 (86.9%)

Note: *N* = 107.

**Table 3:** Relationship between the pre-defined risk factors and the risk of hospitalization and death.

Risk Factors	Risk of Hospitalization and Death		
	*df*	*V*	*p_Fisher_*
Transplantation	1	0.11	0.256
Immunosuppression	2	0.12	0.339
Age > 65 years	1	0.31	**0.003**
Diabetes mellitus	1	0.07	0.552
Obesity	1	0.04	1.000
Chronic obstructive pulmonary disease	1	0.07	1.000
Chronic kidney disease	1	0.07	1.000
Heart failure	1	0.36	**0.001**
Ischemic heart disease	1	0.30	**0.005**
Cardiomyopathy	no cases with cardiomyopathy met		
Nursing home resident	no cases with nursing home resident met		
Active cancer	1	0.17	0.112

Note: *N* = 107. Results which showed statisticaly significant value were marked in bold.

**Table 4:** Relationship between vaccination status, medical history of previous COVID-19 infection, risk of hospitalization, and death.

Risk Factors	Risk of Hospitalization and Death			
	*n*	*df*	*φ_c_*	*p_Fisher_*
Previous COVID-19 infection	107	1	0.03	0.675
Full course of vaccination	107	1	0.02	1.000
Booster dose	83	1	0.14	0.320

**Table 5:** Adverse events categorized by risk groups for severe COVID-19.

Risk Factors	Group	Adverse Events of Molnupiravir Treatment, %																		
		Dizziness	Diarrhea		Vomiting		Urticaria	Headache		Nausea		Rash		Weakness		Abdominal Pain		Herpes Zoster	Deterioration of Blood Pressure Control	
**Transplantation**	No	1.9	0		0		0.9	0.9		2.8		0		2.8		1.9		0	0.9	
	Yes	2.8	0.9		0.9		0	1.9		4.7		0.9		11.2		2.8		0.9	0	
Immunosuppression	No	1.9	0		0		0.9	0		2.8		0		2.8		1.9		0	0	
	Yes	1.9	0.9		0.9		0	2.8		4.7		0.9		11.2		2.8		0.9	0.9	
Age > 65	No	0.9	0.9		0.9		0.9	1.9		3.7		0.9		9.3		3.7		0.9	0.9	
	Yes	3.7	0		0.9		0	0.9		3.7		0		4.7		0.9		0	0	
Diabetes mellitus	No	0.9	0.9		1.9		0.9	1.9		2.8		0.9		9.3		3.7		0	0.9	
	Yes	3.7	0		0		0	0.9		4.7		0		4.7		0.9		0.9	0	
Obesity	No	4.7	0.9		1.9		0.9	2.8		6.5		0.9		14.0		4.7		0.9	0.9	
	Yes	0	0		0		0	0		0.9		0		0		0		0	0	
Chronic obstructive pulmonary disease	No	4.7	0.9		1.9		0.9	2.8		7.5		0.9		14.0		4.7		0.9	0.9	
	Yes	0	0		0		0	0		0		0		0		0		0	0	
Chronic kidney disease	No	1.9	0.9		0.9		0	1.9		2.8		0.9		9.3		2.8		0	0	
	Yes	2.8	0		0.9		0.9	0.9		4.7		0		4.7		1.9		0.9	0.9	
Heart failure	No	4.7	0.9		1.9		0.9	2.8		7.5		0.9		10.3		3.7		0.9	0.9	
	Yes	0	0		0		0	0		0		0		3.7		0.9		0	0	
Ischemic heart disease	No	2.8	0.9		1.9		0.9	2.8		6.5		0.9		11.2		3.7		0.9	0.9	
	Yes	1.9	0		0		0	0		0.9		0		2.8		0.9		0	0	
Cardiomyopathy	No cases with cardiomyopathy explored																			
Nursing home resident	No cases with nursing home residents explored																			
Active cancer	No	4.7	0.9		1.9		0.9	2.8		6.5		0	12.1			3.7		0.9	0.9	
	Yes	0	0		0		0	0		0.9		0.9	1.9			0.9		0	0	
Risk factors	Group	Adverse events of molnupiravir treatment (continued)																		
		Appetite increase		Pneumonia		Catheter-related sepsis			Appetite worsening		Worsening of diabetes control				Pruritus		Hypotension requiring hospitalization		Other	
Transplantation	No	0		0		0.9			0		0.9				0.9		0.9		0.9	
	Yes	1.9		0.9		0			0.9		0				0		0		0	
Immunosuppression	No	0		0		0.9			0		0.9				0.9		0.9		0.9	
	Yes	1.9		0.9		0			0.9		0				0		0		0	
Age > 65 years	No	1.9		0.9		0			0.9		0				0.9		0.9		0	
	Yes	0		0		0.9			0		0.9				0		0		0.9	
Diabetes mellitus	No	0.9		0.9		0.9			0.9		0				0		0.9		0	
	Yes	0.9		0		0			0		0.9				0.9		0		0.9	
Obesity	No	1.9		0.9		0.9			0.9		0.9				0.9		0.9		0.9	
	Yes	0		0		0			0		0				0		0		0	
Chronic obstructive pulmonary disease	No	1.9		0.9		0.9			0.9		0.9				0.9		0		0.9	
	Yes	0		0		0.9			0		0				0		0.9		0	
Chronic kidney disease	No	1.9		0.9		0			0.9		0				0		0		0	
	Yes	0		0		1.8			0		0.9				0.9		0.9		0.9	
Heart failure	No	1.9		0.9		0.9			0.9		0.9				0.9		0.9		0	
	Yes	0		0		0			0		0				0		0		0.9	
Ischemic heart disease	No	1.9		0.9		0.9			0.9		0.9				0		0.9		0	
	Yes	0		0		0			0		0				0.9		0		0.9	
Cardiomyopathy	No cases with cardiomyopathy explored																			
Nursing home resident	No cases with nursing home residents explored																			
Active cancer	No	1.9		0.9		0.9			0.9		0				0.9		0.9		0.9	
	Yes	0		0		0			0		0.9				0		0		0	

Note: *N* = 107. Bolded results were statistically significant.

## Data Availability

Data supporting the results reported in the article can be found under the following DOI: 10.17632/dv2ymdrdrs.1.
